# The proteasome cap RPT5/Rpt5p subunit prevents aggregation of unfolded ricin A chain

**DOI:** 10.1042/BJ20130133

**Published:** 2013-07-12

**Authors:** Paola Pietroni, Nishi Vasisht, Jonathan P. Cook, David M. Roberts, J. Michael Lord, Rasmus Hartmann-Petersen, Lynne M. Roberts, Robert A. Spooner

**Affiliations:** *School of Life Sciences, University of Warwick, Coventry CV4 7AL, U.K.; †Section for Biomolecular Sciences, Department of Biology, University of Copenhagen, Ole Maaløes Vej 5, DK-2200 Copenhagen N, Denmark

**Keywords:** AAA-ATPase, chaperone, endoplasmic reticulum dislocation, proteasome, ricin A chain, Rpt subunit, AAA, ATPase associated with various cellular activities, ALLN, *N*-acetyl-L-leucyl-L-leucylnorleucinal, BFA, brefeldin A, cLβ-l, *clasto* lactacystin β-lactone, CP, core particle, DSS, disuccinimidyl suberate, ER, endoplasmic reticulum, ERAD, ER-associated degradation, GdnHCl, guanidinium chloride, Pi1, proteasome inhibitor 1, RP, regulatory particle, RTA, ricin toxin A chain, nnRTA, non-native forms of RTA

## Abstract

The plant cytotoxin ricin enters mammalian cells by receptor-mediated endocytosis, undergoing retrograde transport to the ER (endoplasmic reticulum) where its catalytic A chain (RTA) is reductively separated from the holotoxin to enter the cytosol and inactivate ribosomes. The currently accepted model is that the bulk of ER-dislocated RTA is degraded by proteasomes. We show in the present study that the proteasome has a more complex role in ricin intoxication than previously recognized, that the previously reported increase in sensitivity of mammalian cells to ricin in the presence of proteasome inhibitors simply reflects toxicity of the inhibitors themselves, and that RTA is a very poor substrate for proteasomal degradation. Denatured RTA and casein compete for a binding site on the regulatory particle of the 26S proteasome, but their fates differ. Casein is degraded, but the mammalian 26S proteasome AAA (ATPase associated with various cellular activities)-ATPase subunit RPT5 acts as a chaperone that prevents aggregation of denatured RTA and stimulates recovery of catalytic RTA activity *in vitro*. Furthermore, *in vivo*, the ATPase activity of Rpt5p is required for maximal toxicity of RTA dislocated from the *Saccharomyces cerevisiae* ER. The results of the present study implicate RPT5/Rpt5p in the triage of substrates in which either activation (folding) or inactivation (degradation) pathways may be initiated.

## INTRODUCTION

The eukaryotic 26S proteasome is a multi-component endoproteolytic complex that cleaves most cytosolic proteins, regulating protein turnover and maintaining cellular homoeostasis. With an essentially common architecture across eukaryotes, it comprises a CP (core particle; the 20S proteasome, barrel) of four stacked heptameric rings (two β rings, which encode the proteolytic activities, flanked on each side by an α ring [1,2]) and one or two RPs (regulatory particles; 19S, cap) [3]. The latter are multi-subunit complexes which recognize and unfold ubiquitin-tagged substrates, feeding them into the central cavity of the CP for destruction. Each RP is subdivided into two main structures, the base and the lid. Applying yeast nomenclature, the base of the RP is built from a ring of six individual AAA (ATPase associated with various cellular activities)-ATPase subunits (Rpt1p–Rpt6p), surmounted by three non-ATPase subunits (Rpn1p, 2p and 13p). A lid complex of other non-ATPase Rpn subunits is connected to the base by the linker subunit Rpn10p.

The proteasome is also the terminal destination for ERAD [ER (endoplasmic reticulum)-associated degradation] substrates [4]. These are misfolded or orphan luminal and integral membrane proteins that are recognized, unfolded and then extracted (dislocated) from the ER via E3 ubiquitin–protein ligase complexes [5] that polyubiquitylate their targets, usually on lysine residues. Polyubiquitylation provides tags for recognition by extraction motors and subsequently for the proteasome [6,7]. The extraction motor is principally the AAA-ATPase Cdc48p (yeast and plants)/p97 (mammals) in complex with its ubiquitin-binding co-factors [8–11], but in the yeast *Saccharomyces cerevisiae*, the proteasome subunit Rpt4p of the RP also plays a role in extracting substrates through the ER membrane [7,12].

A number of proteins dislocate from the ER lumen in an ERAD-like manner but avoid proteasomal destruction, instead undergoing post-dislocation refolding to an active conformation in the cytosol [13–16]. For example, some protein toxins traffic into cells to the ER lumen where their catalytic subunits (referred to as A chains) are dissociated from the cell-binding B chains and dislocated to the cytosol [17]. The best-characterized of these is the disulfide-bonded A-B toxin ricin [18]. A hydrophobic patch near the C-terminus of ricin A chain (termed RTA), previously occluded by its partner B chain before reduction of the holotoxin in the ER lumen [19,20], partially enters the ER membrane in a structurally altered form [21]. This suggests that RTA mimics a misfolded membrane protein prior to engagement with ERAD components [12,22,23] to enter the cytosol as a misfolded protein. To be an effective ribosome-inhibiting toxin, a proportion of the dislocated RTA must avoid proteasomal degradation and refold to an active conformation. A dearth of lysine residues in RTA has been proposed to severely limit the number of sites available for polyubiquitylation and hence reduce its proteolysis [24,25]. This has been supported by findings that irreversible inhibition of the proteolytic activities of the proteasome sensitizes mammalian cells to ricin [24,26]. However, when RTA dislocates from the ER of *S. cerevisiae*, there is no measurable proteasomal de-gradation of RTA, even though an interaction with the proteasome via the RP subunit Rpt4p is required for the dislocation event [12]. These conflicting observations raise questions about the role(s) of the proteasome in determining the fate of RTA post-dislocation. Therefore, in the present study, we examined the interactions of RTA with both mammalian proteasomes and mammalian RP, and report that the RP can prevent aggregation of an unfolded RTA prior to recovery of toxin activity.

## EXPERIMENTAL

### Materials

Recombinant RTA was purified as described previously [24]. Human 26S [40% singly capped/60% doubly capped with 19S regulatory subunits (as determined by the manufacturer)] and 20S proteasomes were from Enzo Life Sciences, and human 19S proteasomes were from Caltag Medsystems. Yeast ribosomes were prepared as described previously [27]. Polyclonal sheep antibodies against bovine casein were from Thermo Scientific; polyclonal sheep anti-RTA and anti-Rpt antibodies have been described previously [28]. Bovine milk β-casein and donkey anti-sheep IgG (whole molecule) conjugated to alkaline phosphatase, streptavidin–alkaline phosphatase, fluorescamine and 97% DMAB (borane dimethylamine complex) were from Sigma, and DSS (disuccinimidyl suberate) was from Pierce. BCIP (5-bromo-4-chloroindol-3-yl phosphate)/NBT (Nitro Blue Tetrazolium) colour development substrate was from Promega. Bio-Spin 6 columns were from Bio-Rad Laboratories. Eppendorf protein LoBind 1.5 ml tubes were used in most experiments in order to keep protein adsorption on the tube wall to a minimum.

### Cytotoxicity measurements

HeLa cells were seeded at 1.5×10^4^ cells/well in a 96-well tissue culture plate, allowed to grow overnight and, after washing with PBS, were incubated for 1–8 h with 100 μl of DMEM (Dulbecco's modified Eagle's medium)/FBS containing graded concentrations of ricin, as indicated in the Figures. Subsequently, cells were washed twice with PBS and incubated in PBS containing 10 μCi/ml [^35^S]methionine for 20 min. After washing the cells twice with PBS, labelled proteins were precipitated with three washes in 5% (w/v) TCA (trichloroacetic acid), the wells were washed twice with PBS and the amount of radiolabel incorporated was determined, after the addition of 200 μl of scintillation fluid, by scintillation counting in a Micro-Beta 1450 Trilux counter. For pharmacological studies, cells were treated in the same manner with graded doses of toxin in medium containing solvent vehicle (control) and with toxin dilutions in medium containing both vehicle and pharmacological agent. Agents, their final concentrations and vehicles were Pi1 [proteasome inhibitor 1 (Calbiochem); used at a final concentration of 50 μM with DMSO as a vehicle], the proteasome inhibitor ALLN [*N*-acetyl-L-leucyl-L-leucylnorleucinal (Calbiochem); used at a final concentration of 20 μM with DMSO as a vehicle], the proteasome inhibitor cLβ-l (*clasto* lactacystin β-lactone; used at a final concentration of 20 μM with DMSO as a vehicle), a mixture of the cathepsin inhibitors pepstatin and leupeptin (used at final concentrations of 100 μM and 1 μM respectively with water as a vehicle) and the secretion inhibitor BFA (brefeldin A; used at a final concentration of 10 μg/ml with ethanol as a vehicle). Toxin trafficking times from cell surface to first destruction of ribosomes were measured as described previously [29].

### Denaturation of RTA

RTA was denatured by incubating in 6 M GdnHCl (guanidinium chloride) or 50 mM HCl (pH 2) for at least 2 h at room temperature (20°C). The denatured protein was then rapidly diluted 100-fold or 40-fold, as appropriate, into reaction mixtures. Alternatively, RTA was denatured by incubation at 45°C for 15 min.

### Proteasome proteolytic activity assays

The fluorescent assay was conducted as described previously [30]. Briefly, RTA and casein were methylated to eliminate free amino groups and assayed for proteasome degradation by incubation with 10 nM 26S proteasome in 20 mM Hepes (pH 7.6) containing 4 mM ATP, 10 mM MgCl_2_ and 1 mM DTT in a reaction volume of 30–50 μl, at the indicated temperatures (see Figures). Aliquots (5–10 μl) were removed (see relevant Figures for the time points) and labelled with fluorescamine. Emission spectra were collected in a Photon Technology International spectrofluorometer, using a 5 or 10 nm pathlength cuvette between 400 and 550 nm (2 nm bandwidth) using an excitation wavelength of 370 nm (2 nm bandwidth). In addition, protein degradation was monitored by SDS/PAGE followed by silver staining or immunoblot analysis.

### Competition assay

Reaction mixtures containing 5 nM 26S or 20S proteasomes, the indicated amount of casein (20, 40 or 400 nM), and, when present, 40 nM RTA (either GdnHCl-denatured or native) in 20 mM Hepes (pH 7.6), 2 mM ATP, 1 mM DTT and 2.5 mM MgCl_2_ were incubated at 37°C for the indicated time points, aliquots were removed, added to pre-heated (95°C) reducing SDS sample loading buffer and incubated at 95°C for 5 min. Samples were then subjected to SDS/PAGE and Western blot analysis using anti-casein antibodies and alkaline phosphatase detection.

### Aggregation assay

GdnHCl-denatured RTA was diluted 100-fold to a final concentration of 40 nM RTA and 60 mM GdnHCl into a solution (20 μl final volume) containing none, proteasome complexes (25 nM 26S corresponding to 40 nM 19S RP, 40 nM 20S or 40 nM 19S), or 40 nM BSA in 20 mM Hepes (pH 7.6), 2 mM ATP, 2.5 mM MgCl_2_ and 1 mM DTT. Alternatively, RTA denaturation was achieved by incubation of native RTA, either in the absence or presence of 19S proteasome, at 45°C for 15 min. Samples were then centrifuged at 12100 ***g*** at room temperature for 10 min, and the supernatant was transferred to a fresh tube. The pellet and soluble fractions were then mixed with reducing SDS loading buffer, heated at 95°C for 5 min, and analysed by SDS/PAGE and Western blotting using anti-RTA antibodies.

### RTA activity assay

Samples from the aggregation assay, either before or after removal of aggregates by centrifugation, were assayed for RTA catalytic activity by incubation at 30°C for 1 h with 20 μg of yeast ribosomes in Endo buffer [25 mM Tris/HCl (pH 7.6), 25 mM KCl and 5 mM MgCl_2_]. Ribosomal RNA was extracted, treated with aniline and analysed as described previously [31].

### Chemical cross-linking and biotin transfer

The chemical cross-linker DSS was dissolved in DMSO and added to a solution containing either denatured 35 nM RTA alone (diluted 100-fold from a 6 M GdnHCl stock) or mixed with 35 nM 26S proteasomes in 20 mM Hepes (pH 7.6). Final concentrations of cross-linker in the reaction mixtures were 0, 40, 400 or 4000 nM. After 2 h at 24°C the reaction was quenched by the addition of 1 μl of 50 mM Tris (pH 7.5) followed by incubation at 24°C for a further 30 min. Samples were analysed by SDS/PAGE and immunoblot using anti-RTA antibodies. For biotin transfer, RTA was conjugated with Mts-Atf-LC-Biotin (Thermo Scientific), following the manufacturer's instructions. Denatured RTA–linker-biotin conjugate was mixed with 19S RP, and biotin was transferred to interacting partners following UV illumination, using the manufacturer's recommendations (Thermo Scientific).

### *S. cerevisiae* growth studies

Synthetic media containing standard ingredients and 2% glucose (SD medium, non-inducing), 2% raffinose (SR medium) or 2% galactose (SG medium, inducing) were used throughout. Selected yeast strains, separately transformed with an expression plasmid encoding galactose-inducible ER-targeted RTA or with vector [12], were grown in SR medium, and after washing in water, were diluted to a nominal concentration of 5×10^6^ cells/ml (*A*_600_=0.5) in SG medium. Growth was followed by monitoring absorbance at 600 nm.

## RESULTS

### Proteasome inhibitors sensitize HeLa cells to ricin only after long exposure

Treatment with cLβ-l, an irreversible inhibitor of all three of the proteasome proteolytic activities [32], sensitizes mammalian cells to ricin intoxication by ~2–3-fold when cells are fixed 6–7 h after initial exposure to cLβ-l [24,26] and ~9–10-fold when fixed ~12 h after initial exposure to cLβ-l [33]. This suggests that the majority of dislocating RTA is normally degraded and that inhibition of the proteasome leads to higher concentrations of toxin, thereby sensitizing cells. However, prolonged exposure to proteasome inhibitors also up-regulates the heat-shock protein Hsp70 [34] with which RTA interacts [35], the ubiquitin–proteasome system [36,37] and apoptosis [38]. Thus long exposures to proteasome inhibitors and ricin may not be appropriate for testing the role of the proteasome in the intoxication process.

**Figure 1:**
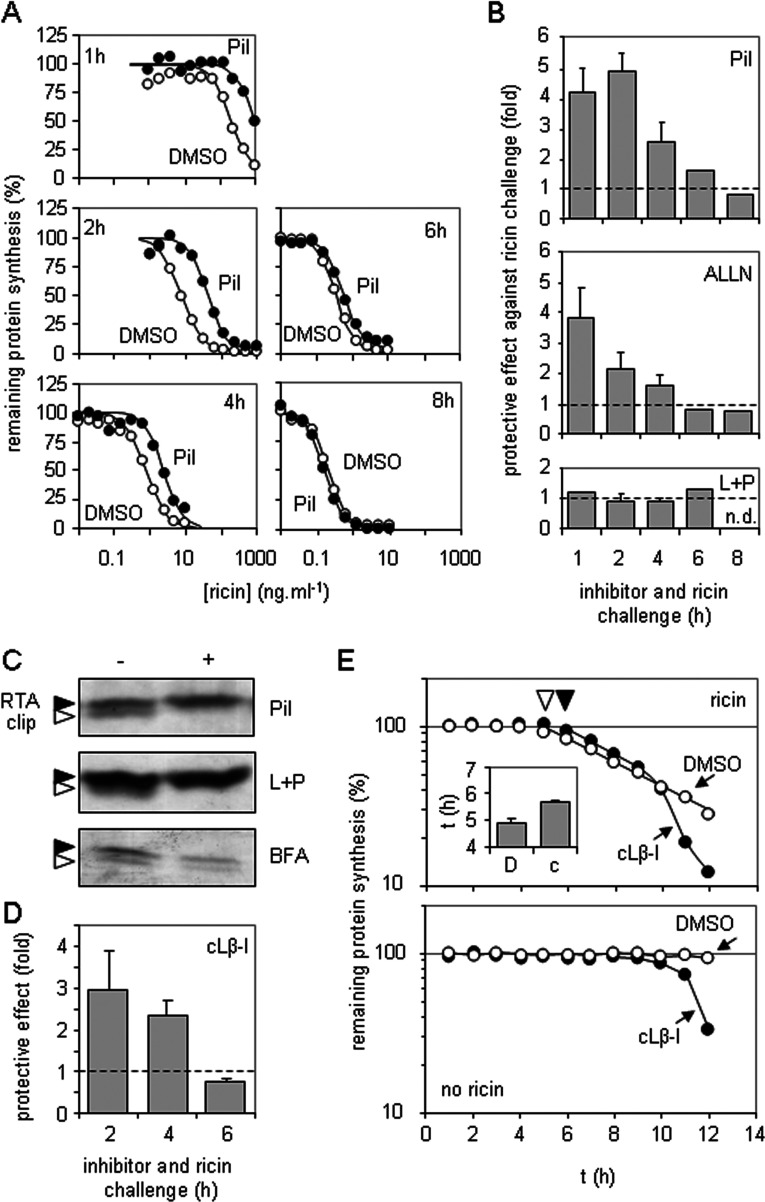
Inhibition of proteasome proteolytic activities sensitizes HeLa cells to ricin only after long incubations (**A**) HeLa cells were treated for 1, 2, 4, 6 or 8 h with graded doses of ricin in growth medium containing 1 μM Pi1 (●) or vehicle (DMSO, ○), and their subsequent ability to synthesize proteins was determined by measuring incorporation of [^35^S]methionine into acid-precipitable material. Typical single assays are shown. (**B**) Top panel: cells were treated as described in (**A**), sensitivities to toxin (IC_50_, toxin concentration required to reduce protein synthesis to 50% that of non-toxin treated controls) were determined, and fold protection (IC_50_ Pi1-treated cells/IC_50_ DMSO-treated cells) is displayed. Middle and bottom panels: cells were treated as described above, substituting the proteasome inhibitor ALLN (middle panel) or a mixture of the cathepsin inhibitors leupeptin and pepstatin (L+P, bottom panel) for Pi1, and substituting water for the vehicle for L+P treatment. Values are means±S.D. for three independent experiments. Broken line, no protective effect over that of treatment with vehicle only; n.d., not determined. (**C**) Cells were treated with a saturating dose of ricin for 4 h in the presence (+) or absence (−) of Pi1 (top panel), L+P (middle panel) or the secretion inhibitor BFA (bottom panel). Detergent-soluble extracts were separated by SDS/PAGE, and RTA (black arrowhead) and a proteolytically clipped RTA (white arrowhead) were revealed by immunoblotting. (**D**) HeLa cells were treated for 2, 4 or 6 h as described in (**A**), substituting the proteasome inhibitor cLβ-l for Pi1. Values are means±S.D. for three independent experiments. Broken line, no protective effect over that of treatment with DMSO only. (**E**) Cells were treated with a single low dose of ricin (1 ng/ml_,_ top panel) for increasing times in the presence of cLβ-l (●) or vehicle (DMSO, ○) and in parallel with cLβ-l (●) or vehicle (DMSO, ○) in the absence of ricin (bottom panel) and their subsequent ability to synthesize proteins was determined by measuring incorporation of [^35^S]methionine into acid-precipitable material. Typical single assays are shown. Inset: toxin trafficking times, determined as in the top panel, in the presence of DMSO (D, ▼) or cLβ-l (c, ▽). Values are means±S.D. for three independent experiments.

We therefore re-examined the effect of proteasome inhibitors in shorter assays than used previously, and found that, in cytotoxicity experiments, the peptide Pi1 protected rather than sensitized cells to ricin. Only in longer incubations were cells sensitized to the toxin (Figures 1A and 1B, top panel). Similarly, the peptide inhibitor ALLN also protected cells from ricin at early time points, sensitizing only at later time points (Figure 1B, middle panel). These proteasome inhibitors also inhibit the major endocytic pathway cathepsins, so we tested the cathepsin inhibitors leupeptin and pepstatin in combination and this time observed little or no effect on ricin toxicity under all assay conditions tested (Figure 1B, bottom panel). These data suggest that the changing protection/sensitization profiles seen with Pi1 and ALLN were a result of proteasome inhibition.

To confirm that the inhibitors used were biologically active we examined detergent-soluble extracts of intoxicated cells to visualize the fate of RTA. In agreement with other studies, in the absence of protease inhibitors, we observed a processing/clipping of the toxin that occurs during intracellular transport of ricin through early endosomes [39,40]. This clipping was inhibited by Pi1 (Figure 1C, top panel) and was also blocked by the endosomal/lysosomal cathepsin inhibitors leupeptin and pepstatin (Figure 1C, middle panel). In the presence of BFA, which traps ricin in the early part of the endocytic pathway [41], clipping remained apparent, confirming that it occurs early in toxin trafficking (Figure 1C, bottom panel). None of the biochemical visualizations performed from inhibitor-treated cells revealed a significant increase in steady-state levels of RTA that would be expected if the toxin was normally dispatched for degradation within proteasomes. Overall, the biochemical experiments support a role for endosomal clipping of RTA *in vivo*, but do not support a role for proteasomal degradation.

Previous reports of sensitization of cells to ricin by proteasomal inhibitors used cLβ-l [24,26,33]. The effect of this compound for short exposures was similar to that of the peptide proteasome inhibitors (compare Figure 1D with 1B). We investigated further by examining a time course of cytotoxicity, treating cells for increasing times with a very low concentration of ricin (to give long incubation times) in the presence of cLβ-l or its vehicle DMSO (Figure 1E) and normalizing each point to coeval controls measuring the effect of inhibitor or vehicle alone. Upon ricin challenge there is a distinct lag phase, which depends on the dose added, followed by an exponential drop in protein synthesis of the cell population [29]. We hypothesized that if the proteasome really does degrade a proportion of dislocating RTA such that cLβ-l treatment results in increased cytosolic recovery of RTA activity [24,26,33], then the kinetics of intoxication would be steeper for cLβ-l-treated cells than for DMSO-treated cells. Instead we saw identical initial intoxication rates (exponential slopes), suggesting there was no proteasomal degradation of dislocating RTA (Figure 1E, top panel); however, there was an increased lag phase prior to intoxication (Figure 1E, inset). This suggests that the protective effect of cLβ-l for short exposures occurred rather unexpectedly from delayed delivery of RTA to the cytosol. Only after extended times (~9 h under these particular conditions) was a very rapid shift from protective effect to apparent sensitizing effect seen, and this transition occurred at the same time as toxicity of cLβ-l treatment alone became apparent (Figure 1E, bottom panel).

We therefore conclude that the reported sensitization of cells to ricin under conditions of proteasomal inhibition describe late events that reflect the toxicity of proteasome inhibitors. It follows that the current model of the role of the proteasome in ricin toxicity needs re-evaluation. Since *S. cerevisiae* Rpt4p interacts *in vivo* with non-ubiquitylated RTA [12], we therefore examined the interactions of mammalian proteasomes with similarly non-ubiquitylated RTA *in vitro*.

### RTA is a poor substrate for proteasomal degradation

**Figure 2:**
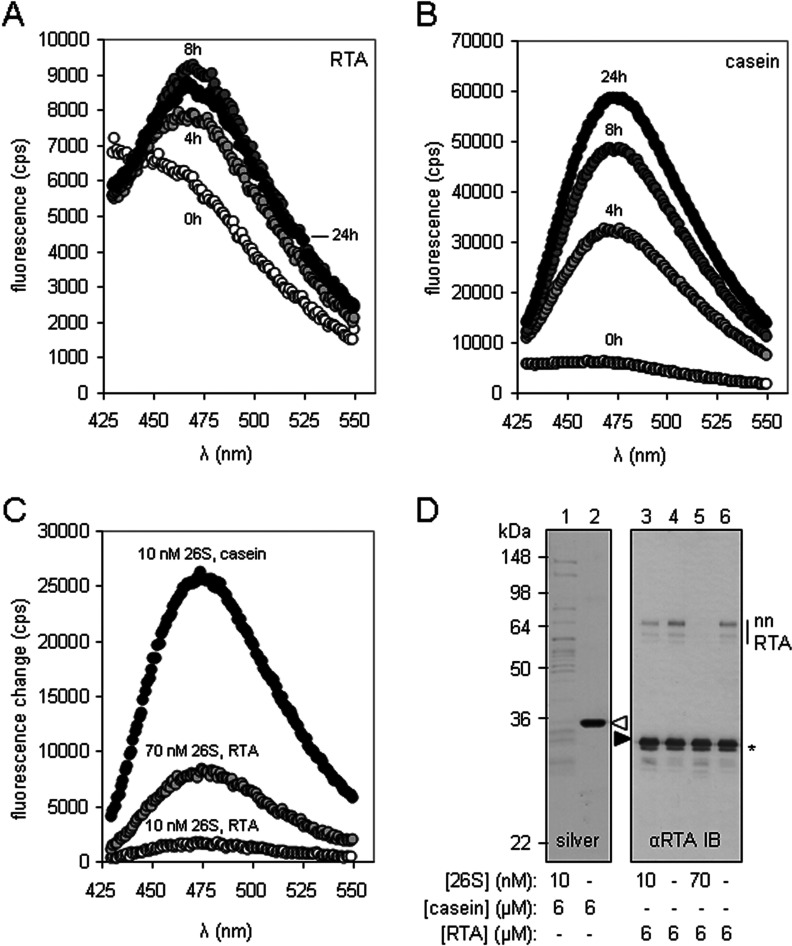
Native RTA is a poor substrate for 26S proteasomes *in vitro* Substrate (6 μM), either methylated RTA (**A**) or methylated casein (**B**), was incubated with 26S proteasomes (10 nM) at 37°C for 4, 8 or 24 h, and (**C**) methylated RTA (6 μM) was incubated at 37°C with 26S proteasomes (either 10 nM or 70 nM) for 16 h, and, in parallel, methylated casein (6 μM) was incubated with 10 nM 26S proteasomes. The extent of fluorescamine incorporation was measured in a spectrofluorometer. (**D**) SDS/PAGE of methylated casein (left-hand panel, silver stained) incubated as described in (**C**), and immunoblot (IB) analysis of methylated RTA (right-hand panel) treated as described in (**C**). Casein, white arrowhead; RTA, black arrowhead. A small fraction of methylated RTA had an increased migration rate in SDS/PAGE (*). Molecular mass in kDa is indicated on the left-hand side.

Native RTA was a poor substrate for 26S proteasomes when assayed by N-terminal fluorescamine labelling of proteolytic fragments generated following incubation with proteasomes (Figure 2A). This contrasts with the behaviour of the well-characterized substrate casein [30] (Figure 2B). Less than 10% of RTA was degraded relative to casein (compare the scales of Figures 2A and 2B). Raising the concentration of 26S proteasomes increased the proportion of RTA degraded (Figure 2C). However, this small degraded fraction of RTA was derived from non-native forms of RTA (nnRTA; Figure 2D, compare lanes 3 and 5) that are probably formed by air oxidation of denatured RTA (R.A. Spooner, unpublished work) and which migrate in SDS/PAGE with a mobility suggesting they are heat-resistant SDS-resistant dimers. There was no obvious degradation of native RTA (black arrowhead, Figure 2D) even in the presence of a large excess of 26S proteasomes. In marked contrast, casein was efficiently degraded (white arrowhead, Figure 2D).

**Figure 3:**
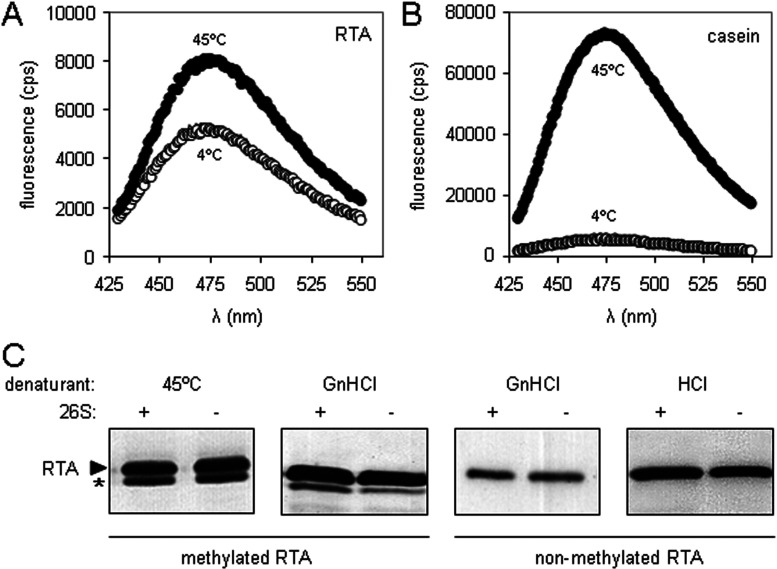
*In vitro* denaturation does not make RTA a better substrate for 26S proteasomes Substrate (6 μM), either methylated casein (**A**) or methylated RTA (**B**), was incubated with 26S proteasomes (10 nM) at 4, 37 or 45°C for 16 h, and the extent of fluorescamine incorporation was measured in a spectrofluorometer. (**C**) Immunoblots of methylated and non-methylated RTA denatured by heat (45°C), chaotrope (6 M GdnHCl) or acid [50 mM HCl (pH 2)], after treatment (+) or not (−) with 26S proteasomes. RTA, black arrowhead. A small fraction of methylated RTA with an increased migration rate in SDS/PAGE (*).

Since a non-native form of RTA (the putative RTA dimer; Figure 2D) can be degraded by 26S proteasomes, we investigated the fate of denatured monomeric RTA. Incubation of proteasome/substrate mixtures at 45°C, which unfolds the thermally unstable RTA [35,42], did not make the toxin a better substrate for proteasomal degradation (Figures 3A and 3B), a result confirmed by SDS/PAGE (Figure 3C, left-hand panel). In the experiment shown in Figures 3(A) and 3(B), both casein and RTA were methylated to block both the N-terminus of the substrates together with any available lysine residues before the addition of proteasomes. This pre-treatment is necessary to reduce background labelling in the fluorescamine assay. To confirm that methylation itself was not responsible for the apparent resistance of RTA to proteasomal degradation, 6 M GdnHCl-denatured methylated RTA and GdnHCl-denatured non-methylated RTA were resolved by SDS/PAGE to reveal no obvious loss of protein (Figure 3C, middle two panels) after incubation with 26S proteasomes. RTA denatured by acid treatment [50 mM HCl (pH 2)] was also refractory to proteasomal degradation (Figure 3C).

### Denatured RTA and casein compete for the 19S RP

**Figure 4:**
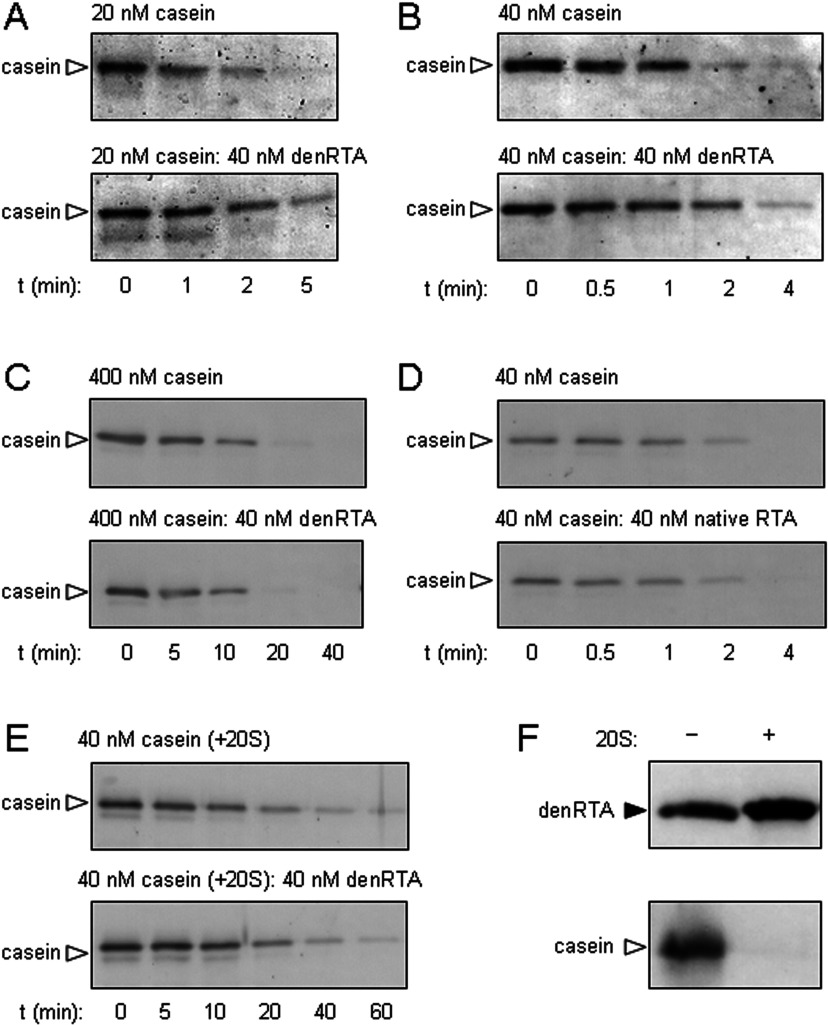
Denatured RTA and casein compete for binding to 26S proteasomes but not to 20S cores The rate of casein degradation is reduced by high concentrations of RTA. Casein (**A**, 20 nM; **B**, 40 nM; **C**, 400 nM) was added to 26S proteasomes (top panels) and incubated (37°C) for various lengths of time. In parallel, reaction mixtures were adjusted by the addition of GdnHCl-denatured RTA (40 nM; bottom panels). Casein fate was determined by SDS/PAGE and anti-casein immunoblot. (**D**) Native RTA does not alter the rate of casein degradation. The experiment was performed as described in (**B**), substituting native RTA for denatured RTA. (**E**) RTA does not influence the rate of casein degradation by 20S proteasomes. The experiment was performed as described in (**B**), substituting 20S proteasome cores for 26S proteasomes. (**F**) RTA is not degraded by proteasome cores. GdnHCl-denatured RTA (40 nM) or native casein (40 nM) were incubated at 37°C overnight in the presence or absence of 6 nM 20S proteasomes, before SDS/PAGE and anti-RTA or anti-casein immunoblot as appropriate. Casein, white arrowhead; RTA, black arrowhead. denRTA, denatured RTA.

In the presence of a 2-fold excess (Figure 4A) or equimolar GdnHCl-denatured RTA (Figure 4B), casein (white arrowhead) was degraded more slowly by intact proteasomes, but in a mixture with a 10-fold excess of casein, no differences in the rates of casein degradation were evident (Figure 4C). In marked contrast, addition of equimolar native RTA had no effect on casein degradation by 26S proteasomes (Figure 4D, compare with 4B). Furthermore, addition of GdnHCl-denatured RTA had no obvious effect on degradation of casein by 20S proteasome barrels (Figure 4E), and denatured RTA was not degraded by 20S proteasomes (Figure 4F). Taken together, these data suggest that denatured RTA and casein compete for the same site on the RP (present in intact 26S proteasomes), but not for a site on the 20S proteasomal barrel.

### The proteasome caps prevent aggregation of denatured RTA

**Figure 5:**
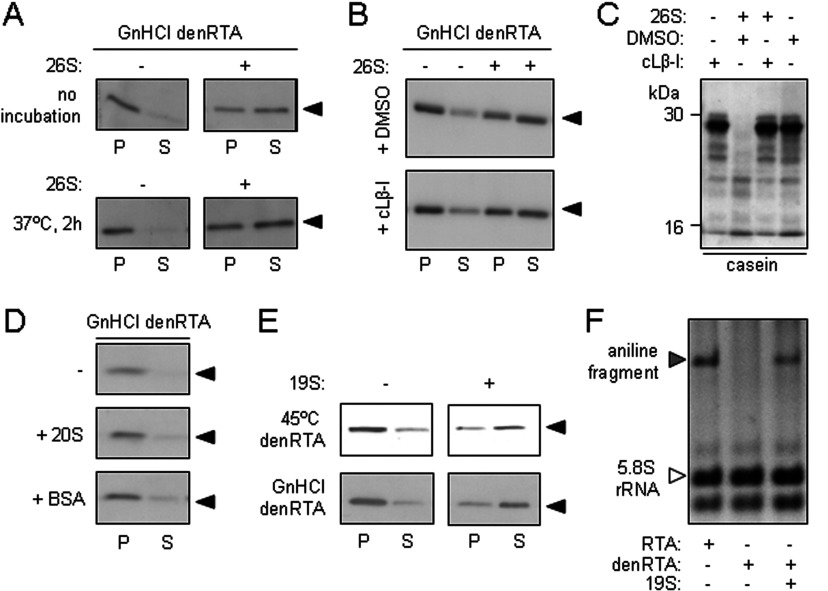
26S proteasomes and 19S proteasome caps are chaperones for denatured RTA (**A**) 26S proteasomes maintain the solubility of denatured RTA. Top panels: GdnHCl-denatured RTA (40 nM) was diluted in the absence (−) or presence (+) of 26S proteasomes (25 nM, equivalent to 40 nM 19S RP) and, after centrifugation, aggregated (P) and soluble (S) fractions were examined by anti-RTA immunoblot after SDS/PAGE. Bottom panels: samples were treated in the same way as above, but were incubated at 37°C for 2 h before SDS/PAGE. (**B**) The solubilizing activity of the 26S proteasome is independent of its proteolytic activity. GdnHCl-denatured RTA (40 nM) was diluted in the absence (−) or presence (+) of 40 nM 26S proteasomes pretreated with the vehicle DMSO (top panel) or the proteasome inhibitor cLβ-l (bottom panel), and aggregated (P) and soluble (S) fractions were examined by anti-RTA immunoblot after SDS/PAGE. (**C**) Efficacy of cLβ-l was confirmed *in vitro* by its ability to block degradation of casein (arrowhead) in the presence of mammalian 26S proteasomes. Casein (40 nM) was incubated in the presence (+) or absence (−) of 26S proteasomes that had been pretreated with vehicle DMSO or cLβ-l. (**D**) 20S proteasome cores do not maintain the solubility of denatured RTA. GdnHCl-denatured RTA (40 nM) was diluted in the absence (−) or presence of 40 nM 20S proteasomes (+ 20S) or 40 nM BSA (+ BSA) and, after centrifugation, aggregated (P) and soluble (S) fractions were examined by anti-RTA immunoblot after SDS/PAGE. (**E**) 19S proteasome RP maintain the solubility of denatured RTA. Heat (45°C)-denatured and GdnHCl-denatured RTA (40 nM) were diluted in the absence (−) or presence (+) of 40 nM 19S RP and, after centrifugation, aggregated (P) and soluble (S) fractions were examined by anti-RTA immunoblot after SDS/PAGE. (**F**) Catalytic activity can be recovered from proteasome-solubilized RTA. Native RTA (RTA), GdnHCl-denatured RTA (denRTA) and a mixture of denRTA and 19S RP were centrifuged to remove aggregates and the soluble fractions were incubated with 20 μg of yeast ribosomes for 2 h at 30°C. After cleavage of any depurinated 28S rRNA with acetic-aniline, rRNAs were extracted, electrophoresed in denaturing conditions (1.2% agarose/50% formamide), and the gel was stained with ethidium bromide for visualization. Aniline fragment, grey arrowhead; 5.8S rRNA, white arrowhead.

After dilution from 6 M GdnHCl, denatured RTA can be separated into an aggregated fraction [P (pellet)] and a soluble fraction (S) by centrifugation. However, when this denatured RTA was diluted by mixing with 26S proteasomes, the proportion of soluble RTA was increased (Figure 5A, top panels), suggesting that the intact 26S proteasome acts as an anti-aggregation chaperone for RTA. Prolonging the incubation of denatured RTA with proteasomes did not increase the proportion of soluble RTA further (Figure 5A, bottom panels). Inhibiting the proteolytic activities of the proteasome by cLβ-l made no obvious difference to the RTA-solubilizing activity of 26S proteasomes (Figure 5B), but it did prevent the degradation of casein (Figure 5C): thus the anti-aggregation properties of the 26S proteasome are essentially independent of its proteolytic activities.

In marked contrast, the 20S proteasome barrel had no overt solubilizing activity for chaotrope-denatured RTA beyond that observed for the control BSA at the same concentration (Figure 5D). This result is consistent with the lack of effect of denatured RTA on the degradation of casein by 20S proteasomes (Figure 4E) and the inability of the 20S CP to degrade denatured RTA (Figure 4F), and suggests that the solubilizing effect of the proteasome on denatured RTA is mediated by the RP. Confirming this, isolated 19S proteasome caps helped to maintain the solubility of both heat- and chaotrope-denatured RTA (Figure 5E). The proportion of denatured RTA solubilized by the 19S RP was greater than that solubilized by 26S proteasomes (compare Figure 5C with 5A), which may suggest that the 20S CP down-regulates or sterically hinders the chaperoning ability of the RP.

RTA is a highly specific N-glycosidase that removes a specific adenine from 26S rRNA in the large ribosomal subunit, thus exposing the rRNA backbone [43]. This site can be cleaved with acetic-aniline (pH 4.5), releasing a small fragment of rRNA that is diagnostic of RTA activity [35]. After incubating native RTA, GdnHCl-denatured RTA and a mixure of denatured RTA and proteasomes, samples were centrifuged to remove any aggregates. The supernatants, representing remaining soluble fractions, were then added to yeast ribosomes. Although ribosome-modifying activity was observed with native RTA, no such activity was seen in the denatured RTA sample, unless the latter had been incubated with RP (Figure 5F). Thus the regulatory caps either promote the folding of denatured RTA to a catalytic conformation or they maintain it in a state that is competent to refold when presented to substrate ribosomes. We were unable to test whether this chaperoning ability is a general feature of the RP with casein, since even after heat or denaturant treatment, we were unable to identify an aggregated population of casein.

### RTA interacts physically with a limited number of RP subunits

After treatment of chaotrope-denatured RTA with the chemical cross-linker DSS, a number of cross-linked adducts were visible (Supplementary Figure S1A at http://www.biochemj.org/bj/453/bj4530435add.htm), with migrations in SDS/PAGE corresponding to that of the nnRTA species from Figure 2(D). In the presence of 26S proteasomes, an extra adduct of ~80 kDa at intermediate concentrations of DSS (grey arrowhead) and a further adduct of ~78 kDa at high concentrations of cross-linker (white arrowhead) became apparent, suggesting that RTA (~29.5 kDa) was cross-linked to two proteasome subunits of ~50 and ~48 kDa respectively. To confirm that interactions of denatured RTA occur with proteasome subunits, we conjugated RTA to Mts-Atf-LC-biotin, a biotin transfer reagent that can be activated by UV illumination, and then denatured the conjugate. Upon UV illumination, in the presence of 19S proteasomes, biotin was transferred from denatured RTA to a ~47 kDa cap subunit (Supplementary Figure S1B, grey arrowhead). The down-regulation of the RP anti-aggregation activity by 20S barrels (Figure 5) suggests the AAA-ATPase RPT base subunits of the RP are candidates for the solubilizing agent(s), since these make direct contacts with the 20S core. Since 47–50 kDa is the approximate size range of the Rpt subunits, we investigated whether or not the ability of the proteasome cap to chaperone RTA resides in these components.

### Mutation of the Rpt3p or Rpt5p Walker A domains permits growth of *S. cerevisiae* expressing ER-dislocating RTA

The conserved architecture of the eukaryotic proteasome, and the existence of defined strains with mutations of the invariant lysine residue in the Walker A motifs of the *S. cerevisiae* Rpt subunits [44], suggests that a functional test for the roles of these subunits can be conducted by measuring growth rates of yeast strains expressing RTA. To this end we expressed a galactose-inducible ER-targeted RTA which subsequently dislocates to the cytosol as a non-native polypeptide in an Rpt4p-dependent manner [12].

**Figure 6:**
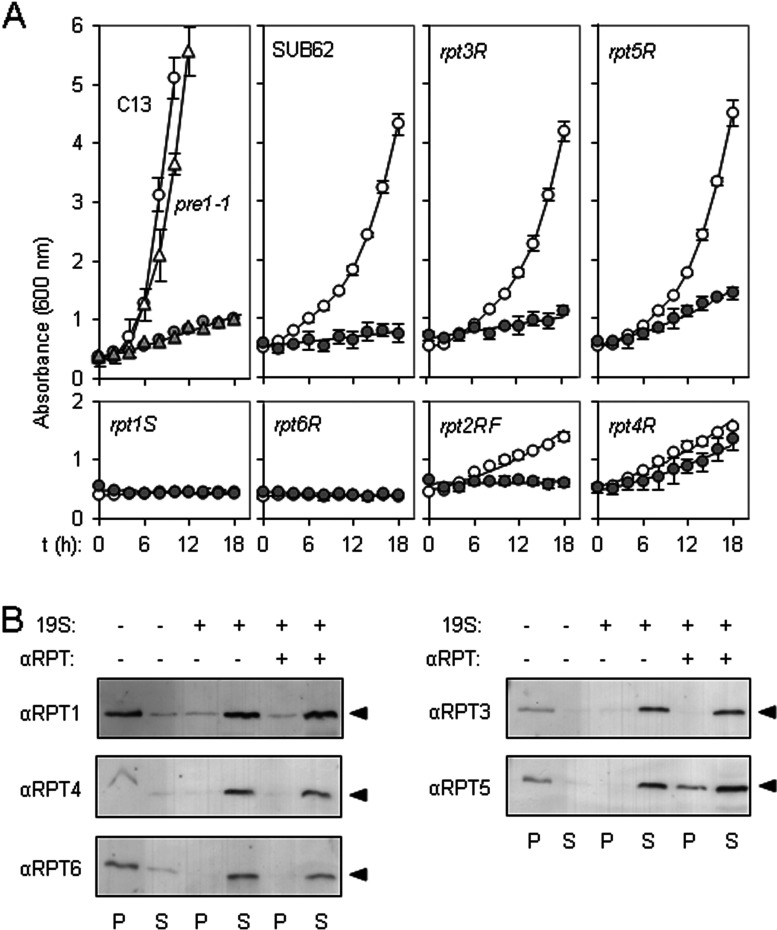
Solubilization of RTA is mediated by the Rpt5 subunit of the RP (**A**) *rpt5R* yeast show resistance to pre-RTA expression. Top left-hand panel: growth in SD-galactose medium of *S. cerevisiae pre1-1* (triangles) and its congenic wild-type strain Cl3-ABYS-86 (C13, circles) transformed with either vector pRS316 (white) or with a pRS316-based expression plasmid encoding preRTA (grey) [12]. Remaining panels: growth in SD-galactose medium of *S. cerevisiae* strains with mutated Walker motifs in Rpt1p, Rpt2p, Rpt3p, Rpt4p, Rpt5p or Rpt6p, and their congenic wild-type strain SUB62 transformed with either vector pRS316 (white circles) or with a pRS316-based expression plasmid encoding preRTA (grey circles). Values are means±S.D. for three independent colonies. (**B**) Anti-Rpt5 antibodies interfere with RP-mediated solubilization of denatured RTA. Denatured RTA (40 nM) was diluted in the presence (+) or absence (−) of 40 nM 19S RP and 160 μg of anti-Rpt1, -Rpt3, -Rpt4, -Rpt5 or -Rpt6 polyclonal serum and, after centrifugation, aggregated (P) and soluble (S) fractions were examined by anti-RTA immunoblot after SDS/PAGE. RTA, black arrowheads.

When tested in the Cl3-ABYS-86 wild-type strain, expression of ER-targeted RTA resulted in a marked growth penalty (Figure 6A, top left-hand panel, compare white circles with grey circles), as a consequence of toxin-mediated ribosomal inactivation (grey circles) [45]. If dislocating RTA were subject to proteasomal degradation *in vivo*, in the congenic *pre1-1* strain defective in proteasome proteolytic functions [46], decreased turnover of RTA would be expected to give a flatter/zero growth rate; however, the growth defect was not increased in this strain (compare white and grey triangles). Thus defects in proteasomal degradation do not have a major influence on RTA toxicity, consistent with there being no obvious degradation of RTA by the proteasome [12].

In comparison, wild-type SUB62 and its *rpt3R* and *rpt5R* derivatives, when transformed with vector, grew equally well (Figure 6A, remaining top panels, white circles), albeit more slowly on galactose medium than C13-ABYS-86. The other strains showed various abilities to grow on the same medium, and the very poor growth of vector-transformed *rpt1S* and *rpt6R* strains in galactose medium precluded meaningful analysis of the effects of RTA expression (Figure 6A, bottom panels, white circles). However, although vector-transformed wild-type SUB62 yeast grew well on galactose, RTA expressed in this same strain caused an immediate severe growth inhibition (grey circles). Consistent with previous findings showing no role for Rpt2p in the fate of dislocating RTA [12], we found that RTA was equally as toxic when expressed in *rpt2RF* cells. In marked contrast, good growth of *rpt4R* cells expressing RTA was observed, consistent with the ER retention of toxin previously noted in this strain [12]. Interestingly, upon RTA induction, *rpt5R* yeast grew reasonably well, suggesting that this proteasome cap subunit normally promotes activity of dislocated RTA *in vivo*. To aid comparison, these data are presented in compiled format lacking symbols in Supplementary Figure S2 (at http://www.biochemj.org/bj/453/bj4530435add.htm).

### Anti-Rpt5 antibodies block the ability of 19S proteasome caps to solubilize denatured RTA

To examine further the role of Rpt5, denatured RTA was incubated in reaction mixtures containing 19S RP in the presence and absence of antibodies against Rpt subunits. Rpt2 was not tested owing to lack of an appropriate antibody. In the absence of competing antibody, RTA was solubilized (Figure 6B), confirming our previous results (Figure 5C); however, in the presence of anti-Rpt5 antibodies, this solubility was reduced (Figure 6B). Thus anti-Rpt5 antibodies block the ability of Rpt5p to chaperone RTA.

## DISCUSSION

The proteasome has a more complex role in ricin intoxication than previously recognized. We had previously shown that *S. cerevisiae* Rpt4p plays a role in extracting non-ubiquitylated RTA (an ERAD-mimetic substrate) from the ER, in a manner that is independent of Cdc48p and its ubiquitin-handling co-factors [12]. This contrasts with *bona fide* ERAD substrates which require Rpt4p in conjunction with ERAD-enabled Cdc48 complexes for cytosolic entry and proteasomal targeting [7]. Furthermore, we could discern no effect of inhibition of the proteolytic activities of the proteasome, nor could we find a role for the Rpt2p proteasome subunit [12], which is required for entry of a substrate into the catalytic core of the proteasome barrel [47]. The interpretation of these observations in yeast clashes with the currently accepted model of the role of the proteasome in ricin intoxication of mammalian cells. This posits that the bulk of dislocated RTA is degraded in a manner that is countered by use of proteasome inhibitors, thereby sensitizing cells to ricin challenge [24,26]. These discrepancies led us to examine in more detail the likely role of the proteasome in ricin intoxication.

To our surprise, the previously reported sensitization of mammalian cells to ricin (repeated in the present study) was seen only when cells had been treated with proteasome inhibitors for longer than ~6 h; shorter periods of inhibition, by contrast, protected cells from ricin by delaying ER dislocation. Kinetic analyses showed that sensitization only occurred when the toxic effects of the inhibitors themselves became apparent. Thus the simple model that the bulk of dislocated RTA is normally degraded in proteasomes may be incorrect, a view now consistent with our findings in yeast [12].

Since only a tiny proportion of incoming toxin reaches the ER, accurate visualization of the amount of RTA that enters the mammalian cytosol is fraught with difficulties [35]. As a starting point, to dissect the role of the proteasome we therefore used defined components to examine proteasome–RTA interactions *in vitro*.

Denatured RTA can be solubilized *in vitro* by the proteasome, and in particular by the RP, consistent with previous reports that the RP can inhibit the aggregation of denatured insulin, denatured citrate synthase and the misfolded nucleotide-binding domain of CFTR (cystic fibrosis transmembrane conductance regulator) [48,49]. Furthermore, RTA activity can be recovered from this solubilized state, again consistent with proteasome-mediated gain of catalytic activity from denatured citrate synthase [49]. Thus denatured RTA belongs to a class of proteins for which the proteasome is a *bona fide* chaperone *in vitro*, resulting in recovery of catalytic activity from a denatured state.

As for denatured citrate synthase [48], the proportion of denatured RTA solubilized by the proteasome caps was greater than that solubilized by 26S proteasomes, pointing to the 20S barrel down-regulating or sterically hindering the anti-aggregation activity of the RP. In turn, this implicates the AAA-ATPase RPT base subunits of the RP, which are in direct apposition to the 20S core, as candidates for the solubilizing agent(s). Consistent with this, denatured RTA can be cross-linked to RP proteins of the size of RPT subunits (~50 kDa), and the anti-aggregation activity of the RP can be inhibited by anti-Rpt5 antibodies.

Allocation of functions to the individual proteins of the proteasome RP is incomplete. Previous views suggested that the lid subunits were required for substrate recognition and ubiquitin release, whereas the base activities were confined to substrate unfolding and translocation into the CP. These have now been modified by findings that the base subunit RPN13 [50,51] and the linker subunit RPN10 [52,53] bind ubiquitin. In addition the base subunit RPT5 has been cross-linked with a tetra-ubiquitin construct [54]. Rpt5p has no obvious role in the dislocation of non-ubiquitylated RTA in *S. cerevisiae* [12], nor in the dislocation of (ubiquitylated) CPY* [7], but it evidently plays a role in the recovery of a catalytic conformation of ER-dislocated RTA in the cytosol; abrogation of its ATPase activity results in a reduction in the toxicity of RTA, supporting a view that RPT5/Rpt5p acts as a chaperone *in vivo*. RPT5 appears to have a binding site for at least two diverse substrates, but the ultimate fate of each substrate differs. *In vitro*, casein is degraded, whereas RTA is solubilized, and, *in vivo*, RTA ultimately gains a catalytic conformation. Thus both substrate inactivation (degradation) and activation (folding) pathways can proceed from the RPT5-bound state. The findings of the present study suggest that RPT5 can parse substrates for proteasomal release or destruction independently of substrate ubiquitylation status.

## Online data

Supplementary data
