# Supplementary material for: The proteasome cap RPT5/Rpt5p subunit prevents aggregation of unfolded ricin A chain
Source: Biochem J. 2013 Jul 12;453(Pt 3):435–45. doi: 10.1042/BJ20130133 (PMC3778710; doi:10.1042/BJ20130133)
Supplement: Supplementary data [file bj4530435add.pdf]

BJ Cell

Biochem. J. (2013) **453**, 435–445 (Printed in Great Britain) doi:10.1042/BJ20130133

SUPPLEMENTARY ONLINE DATA

The proteasome cap RPT5/Rpt5p subunit prevents aggregation of unfolded ricin A chain

Paola PIETRONI*, Nishi VASISHT*, Jonathan P. COOK*, David M. ROBERTS*, J. Michael LORD*, Rasmus HARTMANN-PETERSEN†, Lynne M. ROBERTS* and Robert A. SPOONER*[1]

*School of Life Sciences, University of Warwick, Coventry CV4 7AL, U.K., and †Section for Biomolecular Sciences, Department of Biology, University of Copenhagen, Ole Maaløes Vej 5, DK-2200 Copenhagen N, Denmark

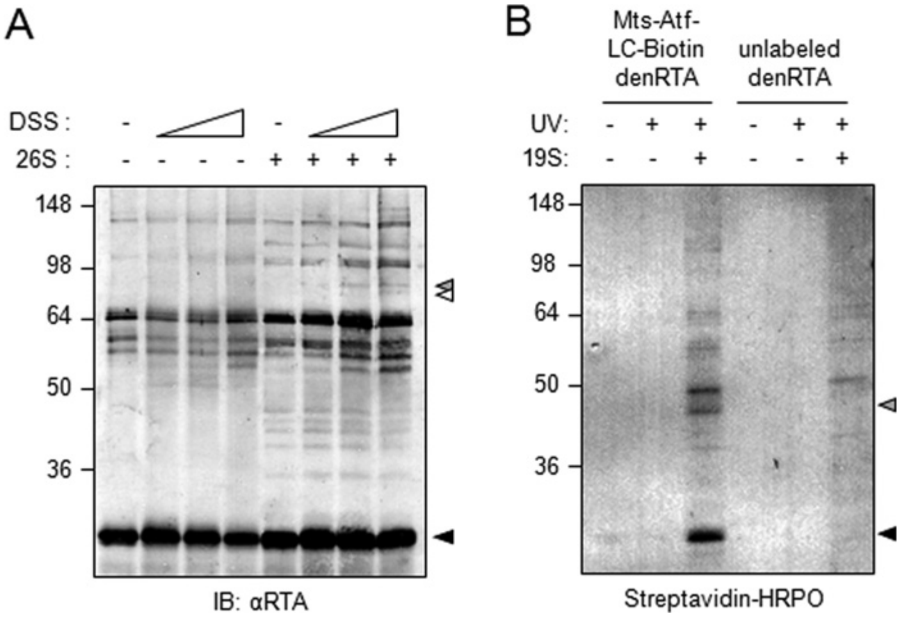

Figure S1 Chaotrope-denatured RTA makes limited interactions with proteasomes

(**A**) GdnHCl-denatured RTA (40 nM) was mixed with (+) or without (−) 26S proteasomes (40 nM) in the absence or the presence of increasing concentrations (40, 400 and 4000 nM) of the cross-linking agent DSS, products were separated by reducing SDS/PAGE and RTA (black arrowhead) and cross-linked adducts (grey and white arrowheads) were identified by anti-RTA immunblot analysis. (**B**) Denatured RTA conjugated with Mts-Atf-LC-biotin was irradiated with UV or mixed with 19S caps and subsequently irradiated, and biotin was identified following SDS/PAGE and blotting by probing with streptavidin. IB, immunoblot; denRTA, denatured RTA. Molecular mass in kDa is indicated on the left-hand side.

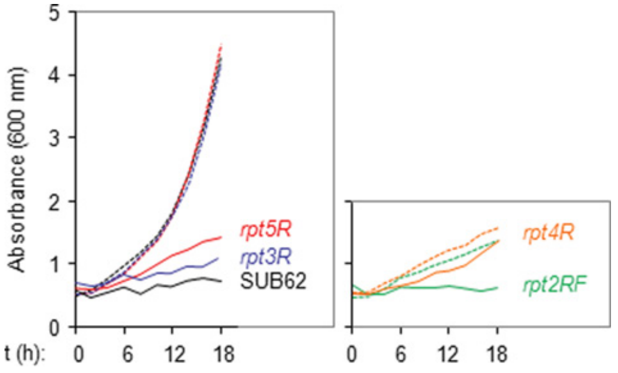

Figure S2 Compiled yeast growth data on galactose medium from Figure 6 of the main text

Curve fits and data points have been removed for clarity. Broken lines, vector transformed; solid lines, RTA-expressing. Black, SUB62; green, rpt2RF; blue, rpt3R; orange, rpt4R; red, rpt5R.

Received 24 January 2013/18 April 2013; accepted 26 April 2013
Published as BJ Immediate Publication 26 April 2013, doi:10.1042/BJ20130133

[1] To whom correspondence should be addressed (email R.A.Spooner@warwick.ac.uk).

© 2013 The Author(s)
The author(s) has paid for this article to be freely available under the terms of the Creative Commons Attribution Licence (CC-BY) (http://creativecommons.org/licenses/by/3.0/) which permits unrestricted use, distribution and reproduction in any medium, provided the original work is properly cited.

Biochemical Journal www.biochemj.org
